# Current Advances and Applications of Animal Models in SARS-CoV-2 Pathogenesis and Vaccine Development

**DOI:** 10.3390/microorganisms13092009

**Published:** 2025-08-28

**Authors:** Li Wu, Yingying Tao, Xing Wu, Shaozhen Li, Rui Yang, Chengying Li, Yao Yao, Shijia Xu, Jianhong Shu, Yulong He, Huapeng Feng

**Affiliations:** 1 Department of Biology, College of Life Sciences, China Jiliang University, Hangzhou 310018, China; y18867136783@outlook.com; 2 Department of Biopharmacy, College of Life Sciences and Medicine, Zhejiang Sci-Tech University, Hangzhou 310018, China; taoyingying20011009@gmail.com (Y.T.); xingtuotuo@foxmail.com (X.W.); lsz2024@yeah.net (S.L.); lcylcylcy111@outlook.com (C.L.); yao3058016703@sina.com (Y.Y.); xushijia_jia@sina.com (S.X.); shujianhong@zstu.edu.cn (J.S.); heyl79@zstu.edu.cn (Y.H.)

**Keywords:** progress, SARS-CoV-2, animal models, pathogenicity, vaccines

## Abstract

COVID-19 is the most widespread emerging infectious disease in humans, recently caused by the SARS-CoV-2 virus. Understanding the pathogenesis and development of efficient vaccines is crucial for the prevention and control of this emerging disease. SARS-CoV-2 viruses have widespread hosts, including humans, domesticated/companion animals (cats, dogs), specific farmed animals (minks), specific wildlife (white-tailed deer), and laboratory animal models. Bats are considered the original reservoir, and pangolins may be important intermediate hosts. Suitable animal models play an important role in studying the pathogenicity and evaluation of vaccines and antiviral drugs during the preclinical stage. In this review, we summarized the animal models and potential animal models for the research of SARS-CoV-2 pathogenesis, vaccine and antiviral drugs development, including transgenic mice, cats, hamsters, nonhuman primates, ferrets, and so on. Our summary provides the important information to select the animals used for a specific purpose and facilitates the development of novel vaccines and antivirals to prevent and control COVID-19.

## 1. Introduction

Global health faces a significant challenge from emerging diseases. Since late 2019, a novel coronavirus named SARS-CoV-2 has emerged to infect humans with severe pneumonia, resulting in death in some cases. As it spread almost all over the world, the World Health Organization (WHO) announced the emergence of a worldwide pandemic. Vaccination and antiviral drugs are two important strategies to prevent and control infectious diseases. As a novel virus, the study of pathogenesis, the development of vaccines, and antiviral drugs is in progress around the world. In this case, animals are critical for these studies mentioned above. In this review, we focused on summarizing the characteristics of the animal models for the research on SARS-CoV-2.

## 2. Virus and Disease

SARS-CoV-2 belonged to the Coronaviridae family, the Betacoronavirus genus. SARS-CoV-2 carries a non-segmented RNA genome with positive polarity. Coronaviruses contain multiple members distributed broadly among humans, other mammals, and poultry [1], seven of which can infect humans, including HCoV-229E, -OC43, -NL63, -HKU1, SARS-CoV, MERS-CoV, and SARS-CoV-2. HCoV-229E, -HKU1, -NL63, and -OC43 viruses typically cause the common cold in children, the elderly, or immunocompromised people [2]. Both SARS-CoV and MERS-CoV cause zoonotic spillover and cause severe respiratory syndrome with high mortality in humans [3]. SARS-CoV-2 infected humans and multiple animals and caused fever, cough, and severe pneumonia since the end of 2019. It spread all over the world, causing a global pandemic. This virus evolved rapidly from alpha to Omicron and then JN.1/KP.2 [4,5].

## 3. Target Cells and Receptors

The target cells of SARS-CoV-2 include macrophages, type II pneumocytes in the lungs, absorptive enterocytes in the intestine, and goblet secretory cells in the nasal passages [6]. Co-expression of ACE2 (Angiotensin converting enzyme II) and TMPRSS2 (transmembrane protease, serine 2) TMPRSS2 within these cells enables these target cells to have a high-level binding ability and demonstrates elevated SARS-CoV-2 susceptibility. SARS-CoV-2 hijacks ACE2 on these host cells as its receptor. Spike (S) protein on the viral surface mediates cell attachment and membrane fusion. During this process, the trimeric S protein is activated mainly by TMPRSS2 and cleaved into S1 and S2 proteins. S1 enables viral attachment through the receptor binding domain (RBD)-ACE2 binding, whereas S2 mediates viral-cell membrane fusion [7].

ACE2 is a type I membrane protein, widely expressed on the alveolar monocytes, macrophages, endothelial cells of arteries and veins, mucosal cells of the small intestine, and epithelial cells of the kidney, renal tubules, alveoli, trachea, bronchial branches, and serous glands [8,9]. In addition to the above, its primary physiological function is to facilitate the maturation of angiotensin (Ang) to control vasoconstriction and blood pressure. For membrane trafficking, ACE2 also functions as a chaperone of the amino acid transporter B^0^AT1 (SLC6A19), which orchestrates neutral AA transport across enterocyte membranes by a Na^+^-leucine cotransport manner [10]. ACE2, in concert with TMPRSS2 as the main host protease, facilitates the virus entry into target cells. Co-distribution of ACE2 and TMPRSS2 in the respiratory tracts is significantly associated with enhanced cellular permissibility to SARS-CoV-2. The animals, including cats, Syrian hamsters, and white-tailed deer, with the highest level of co-expression of these two host factors, are most susceptible to SARS-CoV-2 infection [11]. ACE2 is the main receptor of SARS-CoV-2 for cell entry. We summarize the key amino acids of ACE2 of different animals interacting with the RBD of the S protein, which may partially interrupt the different susceptibility of multiple animal models (Table 1).

Other receptors and host proteases also exacerbate the pathogenesis of SARS-CoV-2, but their modes of action remain unknown, such as neuropilin-1, furin, and tyrosine-protein kinase receptor UFO (AXL), kidney injury molecule (KIM1), and Toll-like receptor 2/4 (TLR2/4) [12,13,14,15]. ASGR1 and KREMEN1 were two alternative functional receptors for non-ACE2 virus entry and further determined the viral tropism and pathogenesis [16]. CD147 functions as a compensatory entry receptor for SARS-CoV-2 in ACE2-deficient cells, which partially explains why lungs can be widely infected by SARS-CoV-2, although ACE2 is expressed at low levels [17]. Interestingly, *leucine-rich repeat-containing protein 15 (LRRC15)*, which was one *TLR*-related cell surface receptor, was found to be a novel receptor of the spike protein of SARS-CoV-2; however, it sequesters the infection of this virus [18]. In addition, integrin contributes to the invasion of SARS-CoV-2 into the non-ACE2 respiratory cells to facilitate its infection and spread in the body of the patients [19]. On the other hand, the S mutant also contributes to the expansion of the host range of the SARS-CoV-2 infection. The S N501Y potentially increases the ability of SARS-CoV-2 to infect avian animals, while the S T478I helps SARS-CoV-2 to utilize multiple mammalian ACE2 for infection [20]. Above all, the binding affinity between the RBD of the S protein and ACE2 determines the permissibility of cross-species (Table 1). And S mutations in the RBD region in Omicron variants enhance the affinity of S to ACE2 of multiple animals; therefore, Omicron has a broader host range [21].

## 4. Animal Models

### 4.1. Pathogenesis of SARS-CoV-2 in Mouse Models

Mice are the preferred small animal model because of the low cost, high reproduction rate, and ease of handling. However, SARS-CoV-2 does not replicate efficiently in normal wild-type laboratory mice because it cannot use mouse ACE2 as the entry receptor [22,23]. In this case, many teams have made great efforts to develop different strains of mice susceptible to SARS-CoV-2. Therefore, different types of transgenic mice have been developed (Table 2).

The first strategy is to transfer the human Ace2 gene to mice and generate the humanized ACE2 transgenic mice. K18-hACE2 mice are the most popular animal model in the research of pathogenesis, vaccines, and antiviral therapy development. K18-hACE2 mice expressed the human ACE2 under an ectopic cytokeratin promoter. hACE2 knock-in (KI) mice with hACE2 expressed under the native promoter were generated. SARS-CoV-2 caused the respiratory infection but not severe diseases in KI mice [24].

A mouse model has been established by introducing human ACE2 (hACE2) for the SARS-CoV-2 receptor into the genome of ICR mice, and the hACE2 transgenic mice have been demonstrated to support SARS-CoV-2 infection [78]. Sun et al. have generated a mouse model expressing hACE2 under the mouse ACE2 promoter by CRISPR/Cas9 knock-in technology. Human ACE2-transgenic murine models are permissive for SARS-CoV-2 upon intranasal delivery and display interstitial pneumonia after being challenged with the SARS-CoV-2 strain BetaCoV/Wuhan/AMMS01/2020 [79]. However, the SARS-CoV-2 was not lethal to the two transgenic mice above. The other transgenic mice have been generated to express hACE2 under the control of Hfh4 (also known as Foxj1) promoter in C3B6 mice, resulting in lethal SARS-CoV-2 infection [25]. The Hfh4 promoter targets hACE2 expression to respiratory ciliated cells and CNS neurons [26]. Hfh4-driven hACE2 transgenic mice exposed to SARS-CoV-2showed about 40% mortality, probably due to viral invasion of the brain [27]. Although transgenic mice displayed interstitial pneumonia, they were not widely available. It takes time to breed enough mice for high-throughput drug and medical development. For some distinct comorbidities, such as diabetes or obesity, transgenic mice cannot meet these needs completely. In addition, human CD147 transgenic mice could also support the infection of SARS-CoV-2, while wild-type mice did not [17]. The first generation of mouse models susceptible to SARS-CoV-2 was mainly established by ectopic expression of hACE2. Nakandakari-Higa et al. utilized the S82M/F83Y/H353K triple mutations of mACE2 to generate a minimally edited mouse model-Ace2, which is susceptible to multiple SARS-CoV-2 strains, including USA-WA1/2020 and B.1.1.529 (Omicron), benefiting research on the immune responses to sequential infection in mice [28]. Mice with F83Y/H353K mutations in the mouse ACE2 supported the infection of SARS-CoV-2, but the infected mice did not manifest symptoms; in addition, further conditional hybrid CMV-Cre derived Rosa26 hACE2 mice were generated, and these mice were more susceptible to the infection of SARS-CoV-2, with reduced body weight, survival rate, and obvious clinical symptoms. These mouse models facilitate the development of SARS-CoV-2 therapy strategies and the research of long COVID-19 [80]. BA.1/BA.2/BA.5 Omicron sublineages emerged from the end of 2021 and spread globally. These viral strains replicate efficiently in the human upper nasal epithelium, while their pathogenicity in the lungs of the infected K18-hACE2 decreased, but they still caused lung pathological changes in aged mice [81]. These results supported the idea that the old were more susceptible to Omicron than the young. Furthermore, Omicron variant infection in K18-hACE2 caused the brain infection with lymphoid depletion [82]. As SARS-CoV-2 evolves, Omicron variants become weak and poorly permissive to B6.K18-hACE2; however, mice with humanized MHC-I and ACE2 were highly susceptible to infection with Omicron [29]. Another strategy to improve the susceptibility of SARS-CoV-2 is to knock out the antiviral gene in the K18-hACE2 mouse model. The researcher found that interferon-induced transmembrane protein3 (IFITM3) knockout K18-hACE2 (K18-hACE2/IFITM3 KO) mice are more permissive than their parental mice [83]. To avoid the neuroinfection disease resulting from the transgenic K18-hACE2 model, the K18-hACE2 transgene was integrated into the collagen type I alpha chain (COL1A1) locus. The Col1a1-K18-hACE2 mice showed weight loss without neurologic symptoms, and it is suitable for the research of long COVID-19 [30]. Ana-Sosa-Batiz et al. generated a triple gene knock-in mouse model, including hACE2, TMPRSS2, and FCGRT, to evaluate the efficacy of monoclonal antibodies targeting the receptor-binding domain of SARS-CoV-2 and hybrid immunity. They found that the mAbs with long half-life are more effective against Omicron BA.2. Therefore, this triple KI mouse model is a useful tool for the development of mAb therapy against COVID-19 [31].

Replication-defective viruses have been utilized as vectors to carry hACE2 and introduced into BALB/c mice via intranasal inoculation to establish SARS-CoV-2 receptor expression in the lung tissue of mice. Hassan et al. have used replication-defective adenoviruses as the vector to establish SARS-CoV-2 pathogenesis models in conventional murine strains. After being infected with SARS-CoV-2, these mice displayed weight loss and immune cell infiltration. In addition, pneumonia has been observed in these mice when given anti-Ifnar1 antibody [23]. In K18-hACE2 mice, B.1.351/Beta variant was the most pathogenic, while lower viral RNA was detectable in the mice infected with BA.1.1/Omicron, and no symptoms were observed. Among them, B.1.351/Beta and BA.1.1/Omicron can infect the wild-type (WT) mice with transient infection, while B.1 and B.1.617.2/Delta cannot infect them [84]. AAV-hACE2-transduced mice were created by using engineered Adeno-Associated Virus (AAV6.2FF) mediated intratracheal hACE2 gene delivery and SARS-CoV-2 replicates to high titers in the nasal turbinate and lungs, inducing the IgM and IgG antibody response, and modulating cytokine production in the nasal turbinate and lungs [32]. A novel humanized mouse model (hACE2 KI) is generated to study long COVID-19 by investigating the accumulation and longitudinal propagation of the tau protein [85].

A humanized mouse model carrying human lung xenografts was developed and was used to investigate the authentic infection of SARS-CoV-2 and it could replicate efficiently and spread quickly in the infected the lung epithelial cells, suggesting it is a good model to explore the pathogenesis of SARS-CoV-2 and develop antiviral therapy, such as Bafilomycin A1 Ad5-hACE2-transduced NIKO mice (NOD-SCID IL2Rgamma(−/−)) developed SARS-CoV-2-induced pulmonary inflammation with cytokine/chemokine dysregulation, providing a humanized platform for viral pathogenicity research and therapeutic assessment [33]. The tissue-specific mouse model was developed by combining the lung-specific Cre mouse and hACE2 mouse to evaluate the effect of antivirals in vivo and pathogenesis in some specific tissues or organs [86]. Three inducible ACE2 transgenic mice, including AT2, club cells, and ciliated cells, were generated, and SARS-CoV-2 infection caused severe pneumonia in all of these mice. These models allow us to investigate the relationship of specific cell types with the pathogenesis of SARS-CoV-2 [87]. In huCD34(+)-hACE2-NCG mice (hACE2-knock-in immunodeficient NCG reconstituted with human CD34(+) cells), SARS-CoV-2 Beta/Delta variants underwent multi-organ replication—including nasal, pulmonary, and brains—by 72h post-infection., but Omicron strains could not detect in the nasal tissue or brain, these results indicate that antiviral immune response did not result in the lack of neurotropic characteristic of Omicron and we should select the proper SAR-CoV-2 strains and mouse models [34]. NSG mice bearing human lung tissue grafts (NSG-L mice) were found to be susceptible to the infection of SARS-CoV-2. A live virus could be recovered from human lung grafts and other multiple organs, and a human immune response was also observed in these humanized mice [35].

The MVA-vectored prefusion-stabilized SARS-CoV-2 spike vaccine candidate was evaluated in K18-hACE2 mice and in golden Syrian hamsters. The MVA-Spf vaccine candidate induced the production of high-titer antibodies, robust T-cell immunity, and robust challenge protection in both animal models [88]. Walls et al. revealed that neutralizing antibody responses in vaccinated BALB/c mice lacked the breadth and potency against SARS-CoV-2 variants of concern (VOCs) observed in primates or humans across heterologous vaccine platforms. Therefore, we need to be cautious when we analyze the data obtained from the BALB/c mice [89].

The second strategy is to modify the SARS-CoV-2 virus to allow it to replicate in wild-type mice. Therefore, a mouse-adapted SARS-CoV-2 virus has been generated to enable it to infect multiple types of mice. Mouse-adapted SARS-CoV-2 strain has been generated by serial passage in aged mice (9 months) through intranasal inoculation. During serial passages, adaptive mutations have been acquired by SARS-CoV-2, such as N501Y in the RBD of the S protein. The mouse-adapted strain with this mutation has displayed increased infectivity and led to interstitial pneumonia in both young and aged mice [90]. This mutation has been demonstrated to enhance the interaction with the receptor ACE2 and infectivity in humans [91]. Dinnon et al. employed reverse genetics to enhance SARS-CoV-2 spike-mACE2 binding affinity, generating a mouse-adapted chimeric virus (MA-SARS-CoV-2) capable of utilizing murine ACE2 for cellular entry. This recombinant strain exhibited robust replication throughout respiratory tract compartments in BALB/c mice across age groups [27]. In K18-hACE2 mice, mouse-adapted SARS-CoV-2 strain MA10 exhibits compartmentalized pulmonary tropism with accelerated replication kinetics, neurodissemination, and altered pulmonary immune profiles [92]. Serial lung passaging in aged BALB/c and C57BL/6N mice generated mouse-adapted SARS-CoV-2 variants (BMA8, C57MA14). The Q489H RBD mutation confers host adaptation by shifting viral receptor usage from human to murine ACE2 [93]. The mouse-adapted BMA8 strain exhibited age-dependent lethality in BALB/c mice but not C57BL/6N counterparts, with host MHC molecules mediating this pathogenic divergence [93,94]. The mouse-adapted SARS-CoV-2 was lethal to the aged mice due to the activation of TNF signaling [95]. To modify viruses is a fast, relatively simple strategy to meet the requirement for infecting different types of mice, but modification of SARS-CoV-2 must be conducted under a biosafety level 3 laboratory. One strategy is to develop the SARS-CoV-2 pseudovirus that contains the firefly luciferase reporter gene, then assess mAb protection and variant tropism via this model by in vivo bioluminescent imaging [96].

The third strategy is to find special mouse strains that could support the infection and replication of SARS-CoV-2. The severe combined immunodeficient (SCID) mice were found to be susceptible to the SARS-CoV-2 Beta B.1.351 variant, and the viral load is high in the lung on Day 3 post-infection. This animal model did not require the use of mouse-adapted virus strains and transgenic mice, so it is an ideal model to evaluate the effect of antiviral reagents [36]. The North American deer mouse could be infected by SARS-CoV-2, and the California mouse presented clinical symptoms [97,98]. The neonatal mouse model is another candidate model for the study of SARS-CoV-2. Although adult mice did not support the transmission of SARS-CoV-2, a model based on neonatal mice was established, and both the ancestral WA-1 and SARS-CoV-2 variants Alpha (B.1.1.7), Beta (B.1.351), Gamma (P.1), Delta (B.1.617.2), and Omicron sublineages BA.1/BQ.1.1could transmit in this animal model by the contact route [37].

### 4.2. Hamsters

Syrian/golden hamsters have been demonstrated as a highly susceptible model for SARS-CoV-2 infection. Weight loss and moderate bronchi-interstitial pneumonia have been observed after SARS-CoV-2 infection. High virus load has been detected in nasal mucosa, lower respiratory epithelial cells, lungs, oral swabs, and rectal swabs by intranasal inoculation of SARS-CoV-2 [38,39,40]. SARS-CoV-2 is efficiently transmitted to naive hamsters by direct contact or via aerosols [40]. Zhang et al. have found that SARS-CoV-2 is only transmitted by direct contact but not by the airborne route [41]. Lee et al. established respiratory SARS-CoV-2 infection in hamsters via oral inoculation. Compared to intranasal infection, oral inoculation displayed no clinical signs, lower viral load, and mild inflammation in lungs, but a comparable level of virus shedding, which may be attributed to the replication of SARS-CoV-2 in the intestines [42].

The Chinese hamster is also susceptible to SARS-CoV-2 infection with significant body weight loss, bronchitis, and pneumonia after infection. Virus replicates in the upper and lower respiratory tract of the infected Chinese hamster. Compared to the Syrian hamster model, the Chinese hamster features advantages including augmented pathology, compact physiology, annotated multi-omics, and modular toolkits [43]. However, it remains unclear whether SARS-CoV-2 transmits efficiently in the Chinese hamster.

The investigation of impairment of mucociliary transport (MCT) in Syrian hamsters with SARS-CoV-2 infection indicates that its deficiency could be a key factor in predicting the progression of SARS-CoV-2 pathogenesis [99]. The inoculation volume of SARS-CoV-2 by the intranasal route directly affects the severity of disease in the Syrian hamster model [100]. The experimental infection in aging hamsters with a high-fat diet showed that hypertriglyceridemia, age, and gender are three important risk factors for the severity of COVID-19 [101]. The other group found that white adipose tissue (WAT) dysfunction induced by SARS-CoV-2 contributes to the greater severity in the old hamsters [102]. Further study found that pulmonary intravascular macrophages were recruited into lungs after SARS-CoV-2 infection, which may contribute to the severe lung inflammation in a Syrian hamster model [103].

Since the human adenovirus and influenza A virus infection are normally present in humans, coinfection of HAdV-5 or IAV and SARS-CoV-2 has occurred [104,105]. The experimental coinfection in hamsters and ferrets demonstrates that SARS-CoV-2 coinfection with HAdV-5 or IAV exacerbates pulmonary pathology versus monoinfection, especially when IAV and SARS-CoV-2 coinfection result in more obvious clinical symptoms than mono-infection [106]. Therefore, the development of combined vaccines against the HAdV-5 or IAV and SARS-CoV-2 is urgently needed. Omicron variant infection could not induce a robust, sufficient protective immune response, but a heterologous challenge could enhance the immune response for other variants of SARS-CoV-2, and indicates that the heterologous antigens vaccination should be recommended for the prevention of COVID-19 [107]. In addition, cepharanthine showed the prophylactic and therapeutic effects against SARS-CoV-2 infection in a Syrian hamster model [108]. Similarly, Omicron variants presented weak and mild symptoms in hamsters. One BA.4 omicron isolate, which lacks the three amino acids (aa 141–143) in non-structural protein 1, did not cause all the typical symptoms in the Golden Syrian hamster model, despite replicating similarly with other SARS-CoV-2 isolates [109]. SARS-CoV-2 exposure induced more severe pathology in aging hamsters than in young and humanized ACE2 hamsters, and the humanized ACE2 hamsters died from SARS-CoV-2-related meningoencephalitis, indicating this model is suitable for the evaluation of the effect on the central nervous system (CNS) [110]. As we know, anosmia is a common symptom caused by COVID-19. The study in hamsters demonstrates that this symptom is due to damage to the olfactory epithelium. There is a moderately strong correlation between the level of anosmia and the thickness of the olfactory epithelium; therefore, we can use the food-searching behavioral test in a hamster model for screening the various therapeutics for SARS-CoV-2-related anosmia and the development of effective therapy [111].

COVID-19 led to a part of patients with neurocognitive impairments or neurodegeneration in clinical settings. More and more reports demonstrate that SARS-CoV-2 induces persistent microgliosis with concomitant accumulation of hyperphosphorylated Tau and α-synuclein in distal brain regions post-infection in a Golden Syrian hamster model, which may cause long-term disorders, such as Alzheimer’s or Parkinson’s disease [112,113,114]. The researcher confirmed that COVID-19 leads to pathological bone loss in a hamster model, which would be beneficial for the development of related interventions against pathological bone loss [115].

SARS-CoV-2 infection occurred in both male and female hamsters, inducing pulmonary pathology comparable to human COVID-19 cases. The male hamsters presented more severe symptoms and had higher viral shedding in the lungs than the female hamsters, which was consistent with the difference in patients. These results confirmed that the Syrian hamster is a suitable model for studying the pathogenesis of SARS-CoV-2 [44].

Syrian hamsters are not only suitable for the initial infection of SARS-CoV-2 and variant infections, but they could also be used as an animal model for evaluation of the pathogenesis of SARS-CoV-2 reinfection after recovery from the initial infection, especially males [116]. So hamsters are a good animal model to study long COVID-19.

Biosafety level 3 facilities are limited to operate the live SARS-CoV-2; therefore, development of mimic infection platform of SARS-CoV-2 in vivo is of great value for evaluating the vaccine efficacy and antiviral therapy under Biosafety level 2 environment, luciferase-expressing vesicular stomatitis virus (VSV)-based SARS-CoV-2 pseudotyped virus was created, and the lungs were harvested 24–72 h after inoculating the hamsters and luminescence was measured using an in vivo imaging system (IVIS) to evaluate the vaccine efficacy and antiviral drugs [117]. Based on the benefit of animal awareness, the three “R” principles should also be considered in using the Golden hamsters when we evaluate the efficacy of the SARS-CoV-2 vaccine in vivo, and rehoming of animals used for scientific and educational purposes under specific guidelines is highly recommended [118]. From the genome and transcriptome analysis, the Syrian hamster model is superior to other rodents to study the SARS-CoV-2 infection since it possesses several important genes, including ACE2, with high homology to human counterparts [45] (Table 2).

### 4.3. Nonhuman Primates (NHP)

#### 4.3.1. Pathogenesis of SARS-CoV-2 in NHPs

Nonhuman primates are often used as the ultimate translational research model due to their close phylogenetic relationship with humans. SARS-CoV-2 infects Old/New World primates, notably rhesus (*Macaca mulatta*), cynomolgus (*M. fascicularis*), African green (*Chlorocebus sabaeus*) monkeys, and marmosets (*Callithrix jacchus*) [46,47,48,49,50] (Table 2). *Macaca mulatta*, *Macaca fascicularis*, and *Chlorocebus sabaeus* are susceptible to SARS-CoV-2 and displayed similar clinical symptoms and pathological lesions, including respiratory abnormalities, increased temperature, weight loss, pulmonary lesions in chest radiographs/computed tomography imaging, viral shedding, histopathological lesions, thrombocytopenia, Type II pneumocyte hyperplasia, and alveolar septal fibrosis [51,52,53,54]. These three models have also been demonstrated as natural transmission models because SARS-CoV-2 can transmit efficiently in these primates [47,51], which is consistent with the same key amino acids in both the three primate models and humans (Table 1). *Callithrix jacchus* is not susceptible to SARS-CoV-2. Viral genomes can be detected in nasal swabs, throat swabs, anal swabs, and blood after infection, but no clinical signs have been observed in *Callithrix jacchus* [48]. From Table 1, we know that several key amino acids of ACE2 in *Callithrix jacchus* are different from those in humans, which may contribute to this phenotype. However, Thomas et al. have found that *Callithrix jacchus* developed interstitial pneumonitis, accompanied by bronchiolitis after infection. Viral antigen is predominantly localized to alveolar macrophages and AT1 pneumocytes. Pulmonary tissues, tracheobronchial lymph nodes (TBLN), and myocardium exhibited viral RNA co-detected with inflammatory lesions in a subset of animals. Hepatitis manifested in most subjects. Pathogenicity on some species of nonhuman primates, especially New World monkeys, after SARS-CoV-2 infection remains unknown, such as squirrel monkeys (*Saimiri*), and mustached tamarins (*Saguinus mystax*). Collectively, it has been revealed that divergent SARS-CoV-2 susceptibility profiles distinguish Old/New World primates, with Macaca mulatta emerging as the optimal SARS-CoV-2 pathogenesis model. The variants of SARS-CoV-2 Omicron (B.1.1.529) were weaker in rhesus macaques than in hamsters and transgenic BALB/c mice. Rhesus macaques are also a good model for the evaluation of antiviral drugs. Remdesivir could significantly reduce the damage to the lower respiratory tract caused by SARS-CoV-2 infection in rhesus macaques [119]. In addition, black-tailed marmoset (*Mico melanurus*) was first reported with natural SARS-CoV-2 infection in Brazil in 2022 [120]. SARS-CoV-2 infection induced the aggregation of immune cells, including B cells, T cells, follicular dendritic cells, and CD169 macrophages in the lungs of the infected rhesus macaques, with the presence of Pulmonary lymphoid tissue [121].

#### 4.3.2. Regulatory and Ethical Issues in Using NHPs for SARS-CoV-2 Study

The use of nonhuman primates (NHPs) in SARS-CoV-2 research is subject to the most stringent regulatory and ethical scrutiny within biomedical science. This is due to their high cognitive capacity, social complexity, and phylogenetic proximity to humans, which makes them both valuable for translational research and ethically costly [55]. Ethically, the core principle is the application of the 3Rs (Replacement, Reduction, Refinement), mandating that their use is only justified when no alternative model exists and that protocols are rigorously designed to minimize numbers and suffering through advanced analgesia, social housing, and early humane endpoints regulated by National Research Council. Regulatory oversight requires approval from institutional committees (e.g., IACUC), which conduct a strict harm-benefit analysis to ensure compliance with animal welfare laws like the U.S. Animal Welfare Act and the EU Directive 2010/63/EU. Research with the infectious SARS-CoV-2 virus must be conducted at Biosafety Level 3 (BSL-3) or enhanced BSL-3 (BSL-3^+^) to add another layer of regulatory scrutiny to protect animal and public health. The acquisition of NHPs for research is tightly controlled. In the U.S., the Centers for Disease Control and Prevention (CDC) regulates the importation of animals to prevent disease transmission. Their source is also critical, with a preference for purpose-bred animals from regulated breeding colonies rather than wild-caught sources, which is often prohibited [56]. Therefore, using NHPs for SARS-CoV-2 research is legally permissible but is one of the most heavily restricted areas of science. It requires navigating a complex web of animal welfare laws, biosafety regulations, and intense ethical scrutiny centered on the 3Rs.

### 4.4. Transmission in Ferrets

SARS-CoV-2 exhibits cross-species transmission among mammals, including minks, raccoon dogs, felids, ferrets, rodents (hamsters/mice), bats (*Rousettus aegyptiacus*), and cervids [122]. Ferrets and hamsters have been commonly used for investigating the transmissibility of respiratory viruses that infect humans [123,124] (Table 2). SARS-CoV-2 exhibits robust replication in ferret upper airways. Viruses have been detected in the nasal turbinate, urine, feces, soft palate, saliva, and tonsils of the infected ferrets. Some infected ferrets developed fever and loss of appetite. SARS-CoV-2 leads to interstitial pneumonia and acute bronchiolitis. Histopathological examination has revealed lymphoplasmacytic perivasculitis, vasculitis, and accumulation of macrophages and neutrophils occurred in the alveolar septa, alveolar lumina, bronchial lining, and bronchial lumina [57,58]. However, Schlottau et al. have found that no clinical symptoms were observed in infected ferrets, which may be attributed to the different strains used in these studies [59]. SARS-CoV-2 was transmitted to naive ferrets by direct contact effectively and by indirect contact limitedly [58,59], while the efficient transmission of SARS-CoV-2 via respiratory droplets and direct contact was documented by Richard et al. [60]. These differences among these groups might depend on different conditions of the experiments carried out, such as facilities, and equipment, virus strains, sensitivity of detection methods, sampling time, and individual differences in ferrets. A recent study demonstrated that ferrets are a suitable model for transmission studies and evaluation of the efficacy of vaccines and antiviral drugs by both intranasal and aerosol administration [61]. Higher expression level of two key entry factors, ACE2 and TMPRSS2, in aged ferrets leads to greater susceptibility than that in young ferrets [62].

Although the antigens of SARS-CoV-2 could not be detected in the brains of the infected ferrets with mild respiratory diseases, SARS-CoV-2 still had neurovirulent potential, with evidence of increased microglial activation and decreased astrocytic activation status [125]. One study demonstrated that SARS-CoV-2 could survive on the skin or skin-to-skin transference and then the virus infected a naive host by skin-to-oral or skin-to-intranasal routes in the ferret model [126]. In conclusion, ferrets are a good animal model for transmission, evaluating antiviral drugs or vaccines against SARS-CoV-2.

### 4.5. Cats

Cats are susceptible to SARS-CoV-2, and younger cats are more susceptible. SARS-CoV-2 can replicate efficiently and transmit via airborne droplets in cats (Table 2). Viral presence was identified in upper respiratory tissues (nasal turbinates, soft palate, tonsils), trachea, lungs, and small intestine following infection [57]. Halfmann et al. found that SARS-CoV-2 could replicate in cats and could transmit efficiently between infected cats and naive cats via airborne droplets, but no symptoms were observed [63]. These results are similar to pandemic situations in humans. Therefore, cats are another ideal model for evaluating the transmissibility of SARS-CoV-2. A linear DNA vaccine candidate elicits high RBD-specific antibody levels and T cell response; these results indicate that cats are a good model for developing a pet vaccine against SARS-CoV-2 [64]. Delta variant (B.1.617.2) of SARS-CoV-2 could cause severe clinical respiratory disease and pulmonary lesions even with a lower amount of SARS-CoV-2 [65]. However, Omicron BA.1.1 presented lower virulence in cats than that of D164G- or Delta variants [66]. Recently, Park et al. also found that cats are not susceptible to the Omicron variants but are susceptible to SARS-CoV-2 infection [127]. The positive antibodies against COVID-19 are prevalent in cats, and the cat-human cross-transmission occurs in a timely manner all over the world [128,129,130].

### 4.6. Minks

#### 4.6.1. Pathogenesis and Transmission of SARS-CoV-2 in Minks

Minks have been reported to be infected with SARS-CoV-2 from humans on two farms in the Netherlands in April and May 2020 [131]. The studies have demonstrated that the virus might be initially introduced by humans, and then spread to minks, and finally transmitted back to humans, based on using whole-genome sequencing of samples collected from minks and workers of outbreaks on 16 farms [67,68]. American mink experimentally challenged with SARS-CoV-2 exhibited severe acute respiratory disease featuring clinical, radiological, and histological pathology [69]. Shuai et al. demonstrated efficient airborne transmission of SARS-CoV-2 in minks, with robust replication throughout the respiratory tract and severe nasal/pulmonary pathology [70]. The natural SARS-CoV-2 infection causes similar symptoms to those of COVID-19 patients. Immunohistochemistry revealed extensive ACE2 receptor expression in turbinate epithelium, with reduced levels in lower respiratory tract epithelium and alveolar macrophages. Correspondingly, IHC and ISH localized the virus predominantly to nasal turbinate epithelial cells. These results provide expanded knowledge of the pathology and pathogenesis of minks infected with SARS-CoV-2, and minks could be used as a potential animal model to study SARS-CoV-2 infection in humans [71]. Minks presented different manifestations infected by different SARS-CoV-2 clusters and dark-colored males, so minks could be a useful animal model for studying the pathogenesis and transmission of SARS-CoV-2 (Table 2).

#### 4.6.2. Regulatory and Ethical Issues in Using Minks for SARS-CoV-2 Study

The use of minks in SARS-CoV-2 research is subject to strict ethical and regulatory constraints due to their high susceptibility and potential for zoonotic transmission and mink-to-human infection [67,70]. The core ethical framework is the 3Rs principle, mandating the Replacement of minks with alternatives where possible, Reduction in animal numbers through rigorous design, and Refinement of protocols to minimize suffering via enriched housing, analgesia, and early humane endpoints regulated by American Veterinary Medical Association)). AVMA Guidelines for the Euthanasia of Animals. Regulatory oversight requires approval from an IACUC, which performs a harm-benefit analysis to ensure compliance with animal welfare laws. Research must also be conducted under high Biosafety Level (BSL-3) containment to prevent SARS-CoV-2 virus escape, safeguarding both animal welfare and public health [132]. In addition, minks are not typical lab animals. Studies that involve intentional infection of minks are heavily scrutinized by departments of agriculture and environment due to the risk of farm outbreaks and potential establishment of an animal reservoir (USDA). This multi-layered governance ensures that any such research is justified, necessary, and conducted to the highest standards of welfare. In conclusion, conducting SARS-CoV-2 research in minks is a highly regulated activity that sits at the intersection of animal welfare law, biosafety, agricultural security, and profound ethical deliberation.

### 4.7. Other Animals

Fruit bats support limited-duration SARS-CoV-2 infection, exhibiting viral tropism for respiratory mucosa (nasal, tracheal, pulmonary) and bronchial-associated lymphoid tissue (BALT). SARS-CoV-2 transmits limitedly in contact with bats [59]. Wild Mexican free-tailed bats (*Tadarida brasiliensis*) can be experimentally infected by SARS-CoV-2 and orally shed moderate amounts of virus for up to 18 days post-infection [72]. Recently, a coronavirus (HKU5-CoV-2) isolated from a patient was found to use the human ACE2 as the receptor to enter the cells. Following experimental challenge, fruit bats develop self-resolving infection where the virus is consistently detected in the nasal epithelium, trachea, lung alveoli, and peribronchial lymphoid tissue [73] (Table 2).

Dogs presented low susceptibility to SARS-CoV-2, and SARS-CoV-2 did not transmit among dogs. Virus RNA can only be detected in rectal swabs in partially infected dogs [57]. However, Alberto-Orlando et al. found that SARS-CoV-2 could transmit from infected owners to cats and dogs by food sharing [133], which is consistent with the national surveillance in the U.S. [97].

Pigs exhibit susceptibility to multiple porcine coronaviruses, including TGEV (transmissible gastroenteritis virus), PEDV (porcine epidemic diarrhea virus), PRCV (porcine respiratory coronavirus), PHEV (porcine hemagglutinating encephalomyelitis virus), SADS-CoV (swine acute diarrhea syndrome coronavirus), and PDCoV (porcine delta coronavirus) [134]. However, pigs and chickens are not susceptible to SARS-CoV-2. The virus could not be detected in swabs and organs. The sera samples were also negative for virus-specific antibody detection [59]. Pigs are a good model for evaluating the safety and immunogenicity of a SARS-CoV-2 RBD fusion heterodimer vaccine candidate, PHH-1V [135]. Pigs expressing human ACE2 (hACE2) exhibit susceptibility to SARS-CoV-2 infection and manifest clinical disease resembling COVID-19 [136].

Tigers and three lions have been reported to be infected with SARS-CoV-2 from a human at the Bronx Zoo, New York, in 2020. The infected animals developed mild, abnormal respiratory signs. SARS-CoV-2 RNA has been detected in respiratory secretions and/or feces from all the infected animals [137]. Three Asiatic lions were found to be infected with the delta mutant (Pango lineage B.1.617.2) of SARS-CoV-2 in India [138]. In addition, contact tracing investigation indicates that lion-to-human transmission occurred in a zoo setting in Indiana, USA [139]. Gorillas were detected as SARS-CoV-2 RNA positive in the Rotterdam Zoo, The Netherlands [140]. Animals from the families Procyonidae and Viverridae were also found to be infected with the SARS-CoV-2 virus in a zoo, indicating that these animals are also a potential model for understanding the characteristics of SARS-CoV-2 [141].

White-tailed deer are susceptible to SARS-CoV-2 infection. Several studies reported that the white-tailed deer presented seroconversion of SARS-CoV-2 infection [74]. SARS-CoV-2 evolves faster than that in humans in free-ranging white-tailed deer, which is a new risk for humans and other livestock [75]. And serological surveillance demonstrates that the positive rate is up to 17.2% [76]. Human-to-deer and deer-to-deer transmission caused the pandemic in white-tailed deer of SARS-CoV-2 [77]. SARS-CoV-2 has been transmitted to domestic mammals, including minks and deer. Viral circulation in these hosts drives species-specific adaptations: minks acquire NSP9_G37E, Spike_F486L/N501T/Y453F, ORF3a_L219V; deer evolve NSP3a_L1035F. Fortunately, these mutations did not advance the ability of human-to-human transmission [142].

In 2020, three snow leopards (Panthera uncia) in a zoo in Kentucky were found to be infected with B.1.2 lineage SARS-CoV-2, closely correlated with human strains. This virus may transmit from humans to snow leopards, and then animal-to-animal transmission occurs [143]. Efficient transmission of SARS-CoV-2 between white-tailed deer has been documented [144,145].

Although small ruminants like sheep and goats are lowly susceptible to SARS-CoV-2, one serological surveillance by ELISA and neutralization assay demonstrated that sheep or goats can be naturally infected with SARS-CoV-2 [146]. SARS-CoV-2 infection in cattle, sheep, goats, and dromedary camels was also found in Oman by serological assay [147].

## 5. Perspective or How to Choose a Suitable Animal Model for Your Research on SARS-CoV-2

The selection of animal models plays a critical role in the evaluation of the pathogenesis of different SARS-CoV-2 strains, the efficacy of vaccines, and antiviral drugs. SARS-CoV-2 variants, WA1/2020, B.1.617.2/Delta, B.1.1.529/Omicron, and BA5.2/Omicron show different outcomes in different animal models. B.1.617.2 (Delta), WA1, and BA.5.2/Omicron exhibit congruent viral kinetics, pathogenicity, and shedding profiles in hamster models, while B.1.617.2 presented the increased pathogenicity in C57BL/6J mice; therefore, the pathogenesis of SARS-CoV-2 is host-dependent [148]. Combined, the animal models were usually used in the evaluation of the efficacy of the vaccine and the effect of antibodies against SARS-CoV-2. The antibody and antibody cocktails could significantly reduce the viral load in the lungs in a hamster model and a macaque model [149,150]. In addition to the presence of entry receptors, animal body temperature and its fluctuations may play some role in the infection and spreading of SARS-CoV-2 [151].

Future technological progress will position advanced 3D culture models—including pulmonary organoids and microfluidic organ chips—as vital platforms for investigating human respiratory disorders, such as COVID-19. These models are closer to reflecting the real host response induced by SARS-CoV-2 infection in human organs than those in animal models, which is crucial for developing more effective vaccines and drugs [152]. An Ex vivo cell model of the human upper respiratory tract is an alternative to the animal model. One study reported that they infected the ex vivo cell model with Alpha and Omicron BA.1 variants for one month, and they could detect viral RNA throughout the infection [153]. The transgenic cell line models are a good alternative to animals. The researcher generated the transgenic DF1 cell line expressing the human ACE2, TMPRSS2, and SARS-CoV-2, which replicated well in this cell line. In addition, avian DF1 cells are engineered to express feline/golden hamster/caprine ACE2-TMPRSS2 receptor complexes. Pigs or horses support the replication of SARS-CoV-2, consistent with the results in vivo [154]. In April 2025, the FDA and NIH announced plans to reduce and finally eliminate the use of animal experiments in the pharmacy and custom development, replacing them with novel methods, such as tissue chip technology, predictive computational simulations, and next-generation in vitro models.

Alternative models based on human biology offer a more direct and often more ethical path to understanding SARS-CoV-2. These four key complement or replacement strategies could be considered. The first method is to use human organoids, including airway, lung, and intestinal organoids grown from stem cells, to study viral tropism, replication, and host response in specific human tissues. These models recapitulate human disease features better than many standard animal models. The second method is organs-on-chips. For example, we can employ lung-on-a-chip devices to model the human alveolar interface and study SARS-CoV-2 infection, immune cell recruitment, and cytokine storm in a dynamic environment that mimics breathing motions. The third strategy is to use human tissue explants. Specifically, we utilize donated human nasal, bronchial, or lung tissues ex vivo to investigate early infection events and test antiviral drug efficacy in authentic human tissue. The last strategy is to apply advanced in silico models. We can use computational biology to model virus–host protein interactions, predict drug effectiveness, and analyze large-scale human genomic data to identify risk factors. These human-based models complement or replace animal studies by providing faster, more human-relevant data for drug and vaccine candidate screening, and for deciphering the mechanisms of SARS-CoV-2 pathogenesis.

## Figures and Tables

**Table 1:** Comparison of residues in ACE2 involved in contact with RBD of S protein of SARS-CoV-2.

Species	19	24	27	28	31	34	35	37	38	41	42	45	79	82	83	325	329	330	353	354	355	357
Chicken	D	-	T	F	E	V	R	E	D	Y	E	L	N	R	F	E	T	N	K	N	D	R
Chimpanzee	S	Q	T	F	K	H	E	E	D	Y	Q	L	L	M	Y	Q	E	N	K	G	D	R
Chinese hamster	S	Q	T	F	D	Q	E	E	D	Y	Q	L	L	N	Y	Q	G	N	K	G	D	R
Chinese tree shrew	T	E	V	F	K	I	E	E	E	H	Q	L	Q	R	Y	Q	D	K	K	N	D	R
Dog	S	L	T	F	K	Y	E	E	E	Y	Q	L	L	T	Y	Q	G	N	K	G	D	R
Domestic cat	S	L	T	F	K	H	E	E	E	Y	Q	L	L	T	Y	Q	E	N	K	G	D	R
Duck (Mallard)	D	-	M	F	E	V	R	E	D	Y	E	L	N	N	F	E	K	N	K	N	D	R
Ferret	S	L	T	F	K	Y	E	E	E	Y	Q	L	H	T	Y	E	Q	N	K	R	D	R
Golden hamster	S	Q	T	F	D	Q	E	E	D	Y	Q	L	L	N	Y	Q	E	N	K	G	D	R
Greater horseshoe bat	S	L	K	F	D	S	E	E	N	H	Q	L	L	N	F	E	N	N	K	G	D	R
Green monkey	S	Q	T	F	K	H	E	E	D	Y	Q	L	L	M	Y	Q	E	N	K	G	D	R
Guinea pig	F	Q	T	F	E	L	K	E	D	Y	Q	L	L	A	Y	Q	K	N	K	N	D	R
Mink	S	L	T	F	K	Y	E	E	E	Y	Q	L	H	T	Y	E	Q	N	K	H	D	R
Mouse	S	N	T	F	N	Q	E	E	D	Y	Q	L	T	S	F	Q	A	N	H	G	D	R
Norway rat	S	K	S	F	K	Q	E	E	D	Y	Q	L	I	N	F	P	T	N	H	G	D	R
Pangolin	S	E	T	F	K	S	E	E	E	Y	Q	L	I	N	Y	Q	E	N	K	H	D	R
Pig	S	L	T	F	K	L	E	E	D	Y	Q	L	I	T	Y	Q	N	N	K	G	D	R
Rabbit	S	L	T	F	K	Q	E	E	D	Y	Q	L	L	T	Y	Q	E	N	K	G	D	R
Rhesus monkey	S	Q	T	F	K	H	E	E	D	Y	Q	L	L	M	Y	Q	E	N	K	G	D	R
Tree shew	T	D	V	F	K	I	E	E	E	Y	Q	L	Q	R	Y	Q	D	K	N	N	D	R
White-foot mouse	S	Q	I	F	K	Q	E	E	D	Y	Q	L	L	N	Y	Q	E	N	K	G	D	R
White-tail deer	S	Q	T	F	K	H	E	E	D	Y	Q	L	M	T	Y	Q	D	N	K	G	D	R
White-tufted-ear marmoset	S	Q	T	F	K	H	E	E	D	H	E	L	L	T	Y	Q	E	N	K	Q	D	R
Human	S	Q	T	F	K	H	E	E	D	Y	Q	L	L	M	Y	Q	E	N	K	G	D	R

**Table 2:** Summary of advantages and disadvantages of each model for SARS-CoV-2 study.

Animals	Classification	Advantages	Disadvantages	Refs.
Transgenic mice	K18-hACE2, Hfh4-hACE2, CD147-hACE2, mACE2-mutant, hMHCI-hACE2, Colla1-hACE2, hACE2-TMPRSS2-FCGRT, AAV-hACE2, Ad5-hACE2, hCD34(+)-hACE2-NCG, SCID	Low cost and accessible; Ease to handle and genetic manipulation; susceptible; characterize the clinical presentation of COVID-19, including weight loss and interstitial pneumonia or severe pneumonia.	Wild-type not infectious; Time cost for construction; Not transmissible; High viral titers in the brain.	[17,24,25,26,27,28,29,30,31,32,33,34,35,36,37]
Hamster	Syrian hamster; Chinese hamster	Low cost and easy to handle; Susceptible to all VOCs; transmissible; Present age and sex bias associated with clinical disease; no or mild clinical disease symptoms.	Virus clears rapidly and is nonlethal. Related reagents shortage.	[38,39,40,41,42,43,44,45]
Nonhuman Primates	Rhesus, Cynomolgus, African green monkeys, marmosets	Susceptible; Highly similar to humans; Mild to moderate clinical signs; Older age is associated with increased disease severity.	High cost; Shortage; Ethical issues; Almost no severe cases.	[46,47,48,49,50,51,52,53,54,55,56]
Ferret	Ferret	Highly susceptible and transmissible; Mild clinical disease; Aged ferrets lead to greater susceptibility.	Relatively high cost; Difficult to obtain specific reagents.	[57,58,59,60,61,62]
Cat	Cat	Susceptible; Highly transmissible.	Lack of standard cats; Reagent shortage.	[57,63,64,65,66]
Mink	Mink	Highly susceptible and highly transmissible; No to mild signs up to strains;	The lab is difficult to handle.	[67,68,69,70,71]
Others	Fruit bats; dogs; pigs; deer, etc.	Susceptible and transmissible (deer); Permissive (Fruit bats).	Low susceptibility (dogs, pigs); Shortage of standard animals (bats, deer).	[57,59,72,73,74,75,76,77]

## Data Availability

No new data were created or analyzed in this study. Data sharing is not applicable to this article.
